# Performance of the Fall Armyworm, *Spodoptera frugiperda* (Lepidoptera: Noctuidae), over Three Generations on Four Maize Cultivars

**DOI:** 10.3390/insects16070719

**Published:** 2025-07-12

**Authors:** Bo Zhang, Jing Yi, Yan Yan, Yirui Wang, Yana Xue, Haiwang Yan, Meifeng Ren, Daqi Li, Guoping Li, Junjiao Lu

**Affiliations:** 1 Shanxi Key Laboratory of Bioagent Utilization and Eco-Pesticide Innovation, College of Plant Protection, Shanxi Agricultural University, Taiyuan 030031, China; 15133420913@163.com (B.Z.); yijingi2023@163.com (J.Y.); 18135992970@163.com (Y.Y.); 17835878261@163.com (Y.W.); 13810605632@163.com (Y.X.); yhw15245958939@163.com (H.Y.); sxzbsrmf@163.com (M.R.); daqi_li@sxau.edu.cn (D.L.); 2 Institute of Plant Protection, Henan Academy of Agricultural Sciences, Zhengzhou 450002, China; liguoping1976@163.com

**Keywords:** insect–plant interactions, pest control, agricultural entomology, crop protection, control technology

## Abstract

The fall armyworm (FAW), *Spodoptera frugiperda* (J.E. Smith), is a highly destructive pest that poses serious threats and causes significant losses to the production of maize in China. This study evaluated the feeding and oviposition preferences of *S. frugiperda* when reared on four maize cultivars—sweet, waxy, common, and silage—across three consecutive generations. It also compared population adaptability among these cultivars and analyzed population parameters between the F1 and F3 generations. The findings revealed that all four F1 generation populations showed a preference for feeding and oviposition on sweet maize. However, over time, *S. frugiperda* exhibited a stronger preference, in terms of feeding and oviposition behaviors, for the natal host plant across three consecutive generations of rearing. The fall armyworm completed its life cycle and oviposited on all four maize varieties over three generations. The sweet cultivar population had the highest intrinsic rate of increase, finite rate of increase, net reproductive rate, larval survival rate, pupation rate, eclosion rate, fecundity, and pupal weight, while the silage cultivar population had the shortest larval stage, pre-adult stage, and adult lifespan and the pupal weight and the fecundity were the lowest. Overall, the population fitness was the highest on the sweet cultivar, and the lowest on the silage cultivar. Compared with F1, the F3 generation of the FAW had a significantly shorter developmental duration in four maize cultivars. Except for the waxy maize cultivars, the fecundity of the other three cultivars did not differ significantly between F1 and F3. This study provides fundamental information on the trend of fall armyworm population changes in maize fields and serves as a reference for rational maize cultivar planting decisions.

## Simple Summary

The fall armyworm is a major pest that causes severe damage to maize crops in China, threatening food security. This study examined how four cultivars of maize—sweet, waxy, common, and silage—affected pest feeding, egg laying, and population growth over three generations. We found that newly hatched fall armyworms initially preferred sweet maize, but over time, they increasingly favored the cultivar of maize on which they were born. Although the pests could survive and reproduce on all four maize cultivars, their growth and reproduction rates varied significantly. Sweet maize supported the fastest population growth, highest survival rates, and largest egg numbers, whereas silage maize led to the lowest populations, manifested by fewer eggs and smaller pupae. By the third generation, pests developed faster across all maize types. These findings will help farmers choose maize varieties that are less likely to support pest outbreaks—such as silage maize—and avoid high-risk options, such as sweet maize. This knowledge aids in reducing crop losses, protecting maize yields, and safeguarding food production for communities.

## 1. Introduction

The fall armyworm, *Spodoptera frugiperda* (J.E. Smith) (Lepidoptera: Noctuidae), is native to tropical and subtropical regions in the Americas. In December 2018, the FAW was first detected in Yunnan Province, China [1], and quickly spread to 26 provinces, autonomous regions, and municipalities, posing a serious economic threat to China’s agricultural production [2]. The FAW is an obligatory migratory insect with a strong long-distance migration ability, capable of moving hundreds of kilometers between different regions and host plants. It can exist continuously throughout the year in the tropical and subtropical regions of southern China [3]; however, when the temperature rises in spring, it migrates seasonally to the north with the East Asian and Indian monsoons [4]. Due to its lack of diapause capacity and sensitivity to cold, it cannot survive at extremely low temperatures and returns south after autumn [5].

The FAW is a herbivorous pest with a broad host range, including 353 host plant species across 76 plant families, predominantly Gramineae, followed by Compositae and Leguminae [6]. The FAW evolved into two distinct haplotypes in the Americas, with one type primarily utilizing maize (*Zea mays* L.), sorghum [*Sorghum bicolor* (L.)], and cotton (*Gossypium hirsutum* L.), while the other type utilizes rice (*Oryza sativa* L.) and various forage crops [7,8]. Based on *COI* and *Tpi* gene analysis, the invasive Chinese population was identified as the maize type [9]. At the end of 2018, there were 3609 authorized maize cultivars in China, with a planting area exceeding 41 million hectares in 2019 [10]. A total of 1.125 million hectares of crops in China were devastated by the FAW in 2019, resulting in economic losses for the country’s maize production ranging from USD 5.4 to 47 billion annually [11,12]. The FAW prefers maize as its host plant, causing damage at any stage, particularly the young leaves and growth points. Adults prefer maize for oviposition, and their larvae have significantly higher hatching and survival rates than on other plants [2].

Host selection by phytophagous insects is a complex process that involves multiple factors. However, many insects tend to prefer natal plant species (on which they have developed) when selecting plants for feeding or oviposition [13]. Hopkins’ host selection principle (HHSP) states that environmental cues experienced by insects during their early stages influence their behavioral choices in later developmental phases [14]. For example, the feeding preferences of *Perina nuda* larvae are influenced by prior feeding experience, particularly when encountering different host choices [15]. Insects’ prior experiences with host plants can modify their subsequent feeding and oviposition behaviors through learning [16,17]. However, these studies primarily focused on short-term trials (specific developmental stages or a single generation) to explore the effects of host experience on insect preferences. Whether learned behaviors emerge after multiple generations of continuous feeding on the same host, or whether such prolonged feeding experiences induce preference-driven shifts in host selection behavior, has often been overlooked.

Host plant species are an important factor affecting insect population growth and outbreaks. The relative adaptability of FAW populations to different host plants may result in different local population dynamics, and the longevity, fecundity, and survival rates may vary with the host plant on which the larvae feed. The effects of various host plants on FAW fitness and population parameters are detailed in Acharya et al., Li et al., and Lu et al. [18,19,20]. According to various studies, there is a growing distinction in growth and development patterns among successive generations as the number of generations increases [21,22]. However, many experiments only show comparisons between the first generation on different host plants or between different generations feeding on the same variety. Nevertheless, the influence of different maize cultivars on the host plant preference (larval feeding and adult oviposition) and developmental parameters (such as stage-specific lifespan, pupal weight, survival rate, and fecundity) of multiple generations of FAW populations has not been adequately addressed. Therefore, it is necessary to conduct studies on multiple generations of FAW populations to reflect the fitness of the FAW on different cultivars and the different traits among the cultivars. This will result in more precise long-term population predictions and will provide critical data for long-term FAW management.

The age-stage, two-sex life table provides a comprehensive and accurate description of the performance of insect populations under specific experimental conditions [23]. Under laboratory conditions, the age-stage, two-sex life table method was used to analyze the FAW population that feeds on various maize cultivars and has been reared for multiple generations. The F3 generation population’s growth and development indexes (including developmental duration, pupation rate, eclosion rate, and fecundity in the adult stage) on various maize cultivars will be assessed and compared to determine their relative fitness. These research findings offer a basis for making informed decisions on planting various maize cultivars and supply fundamental knowledge for comprehensive FAW management.

## 2. Materials and Methods

### 2.1. Insect

The experimental insects were raised in an artificial climate box at a temperature of 25 ± 1 °C, relative humidity of 65 ± 5%, photoperiod of 16 L: 8 D, and light intensity of 12,000 lux (No. PRX-450C; Ningbo Jiangnan Instrument Factory, Ningbo, China).

Newly hatched larvae were introduced into a plastic insect-rearing box with a calligraphy brush and fed artificial diet (Table A1 for formula component and quantity) [24]. The first- to third-instar larvae were fed in groups, while the fourth- to sixth-instar larvae were individually fed in round plastic boxes (25 mL, bottom diameter 3 cm, top diameter 4 cm, height 3.5 cm). After the larvae pupated, the pupae were collected in a round plastic box covered with sand until emergence, and newly emerged adults were placed in an insect cage (35 cm × 35 cm × 35 cm) for mating. Adults were provisioned with a 15% honey solution, and eggs were collected and placed in a ziplock bag for incubation and hatching.

Insect-rearing boxes, each containing different cultivar maize leaves (sweet, waxy, ordinary, or silage), were provided to newly hatched FAW larvae that had been fed an artificial diet. The larvae of the F1 and F2 generations were reared in groups in the insect box before the third instar and individually reared in a plastic box after the third instar. The FAWs of generation F3 were reared individually during the larval and pupal stages, and a bisexual life table was created. They were observed every 24 h, the insect boxes were cleaned, and leaves were replaced.

### 2.2. Host Plants

The tested maize seeds were sweet cultivar (cv. Jinchaotian), waxy cultivar (cv. Jinxiannuo 6), silage cultivar (cv. Tongli 8), and common cultivar (cv. Dafeng 30). Four different cultivars of maize seeds were planted in plastic pots (50 cm × 35 cm × 25 cm), and substrate nutrient soil was used for planting. The sowing depth was 5 cm, and the planting density was 50 plants per pot. The maize plants were cultivated under natural outdoor lighting conditions. Irrigation was performed at 2- to 3-day intervals. After the maize had grown to the 5-leaf stage, young leaves were cut for feeding and testing. None of the host plants for the test were exposed to any pesticides.

### 2.3. Determination of Spodoptera frugiperda Oviposition Preference

An oviposition preference assay was conducted using a cage experiment to evaluate the oviposition preferences of female fall armyworm adults on four maize varieties. Maize plants of the four varieties at the three-leaf stage with similar plant heights and leaf sizes were randomly arranged at the four corners of a mesh cage (40 cm × 40 cm × 40 cm, 200-mesh). A plastic Petri dish filled with cotton moistened with a 15% honey solution was placed at the center of the cage. Eight newly emerged adult pairs (1:1 sex ratio) were introduced into the cage and allowed to mate freely. The number of eggs deposited on each variety was recorded 48 h post-introduction, with data collected continuously for 5 days across three experimental replicates. Plants were watered daily, and the honey solution was replenished as needed. The formula for calculating the egg attachment rate was as follows: (number of eggs on a single maize variety/total number of eggs across the four maize varieties) × 100%. The entire experiment was conducted in a climate-controlled greenhouse at a temperature of 25 ± 1 °C, relative humidity of 65 ± 5%, and a photoperiod of 16 L: 8 D.

### 2.4. Determination of Spodoptera frugiperda Feeding Preference

Feeding preference was assessed using the leaf disk assay. Plastic Petri dishes (15 cm diameter) lined with filter paper were divided into four equal sections, each containing maize leaf segments of equal weight that were moistened to maintain hydration. Ten second-instar larvae starved for 6 h prior to the assay were placed at the center of each dish. The experiment included ten replicates per treatment. The number of larvae feeding on each variety was recorded after 24 h to calculate the feeding selection rates. The experimental conditions were identical to those described in the Section 2.1.

### 2.5. Construction of Life Tables of Spodoptera frugiperda

Using the laboratory population life table method, we evaluated the effects of continuous multi-generation rearing on different maize cultivars on the growth, development, and reproduction of FAWs. F3 generation eggs that were continuously reared for multiple generations with different maize cultivars were used as the test insects. After hatching, the leaves of the four different maize cultivars were used to feed the larvae. Newly hatched larvae were placed in plastic boxes for individual rearing. In the life table study, a total of 100 newly hatched larvae were used for each maize cultivar, each larva as a replicate. Maize leaves were replaced every day, and larval survival, instar, pupation, eclosion, and other parameters were recorded daily. The pupal weight was measured on the second day after pupation. After emergence, adults of the same day (one female and one male) were placed in a transparent plastic cup (250 mL, with a bottom diameter of 5 cm, a top diameter of 7.5 cm, and a height of 7 cm) for 1:1 pairing. The plastic cup was sealed with double-layer degreasing gauze for female adults to lay eggs. A cotton ball soaked with 15% honey water was hung in the plastic cup for adults to replenish nutrition and was replaced daily. On the second day after mating, egg laying by the adults in the plastic cup was observed and recorded. The eggs were collected daily until the adults died, and the lifespans of male and female adults were recorded. The entire test was conducted in an artificial climate box with a temperature of (25 ± 1) °C, relative humidity of (65 ± 5) %, and photoperiod of 16 L: 8 D.

### 2.6. Life Table Data Analysis

The data for the experiment were collected and analyzed using the TWOSEX-MS Chart 2022 software [25] (available at http://lifetablechi.com/software/, accessed on 9 January 2023). The age-stage-specific survival rate (*s_xj_*), age-specific survival rate (*l_x_*), age-stage-specific fecundity (*f_xj_*), age-specific fecundity (*m_x_*), age-specific maternity (*l_x_m_x_*), net reproductive rate (*R*_0_), intrinsic rate of increase (*r*), finite rate of increase (*λ*), mean generation time (*T*), age-stage-specific life expectancy (*e_xj_*), age-stage-specific reproductive value (*v_xj_*), and other analytical data were processed and plotted [26,27]. All definitions, equations, and references are listed in Table A2.

The mean and standard errors of each parameter were calculated by using the bootstrap method supported by the TWOSEX-MS Chart software (with 100,000 bootstraps), and then the paired bootstrap test was used to conduct significance analysis of differences in the data [28].

### 2.7. Data Processing and Mapping

The original data source was recorded and organized using Excel 2022, and then the original data source was analyzed using the TWOSEX-MS Chart software and plotted using SigmaPlot 15.0 (SigmaPlot Software, San Rafael, CA, USA). Normality of feeding and oviposition preferences were measured using the Kolmogorov–Smirnov test in SPSS v.27.0, then one-way analysis of variance (significance level set at 0.05) was performed, followed by Duncan test comparisons to determine significant differences. Percentage data were converted to the square root of the arcsine before one-way analysis of variance (ANOVA) to meet the requirements of ANOVA.

## 3. Results

### 3.1. The Oviposition Preference of Spodoptera frugiperda on Four Maize Cultivars for Three Consecutive Generations of Feeding Experiences

The oviposition preference of the FAW populations reared for three consecutive generations on the same maize cultivar exhibited significant changes (Table 1). The F1 generation predominantly oviposited on sweet maize in all populations, but their preference gradually shifted toward the original maize cultivar that they had previously fed on as generations progressed. For instance, in the sweet cultivar and silage cultivar populations, significant differences in egg-laying rates on the original maize cultivar were observed between the F1 and F3 generations (*F* = 6.00, *d.f.* = 2,6, *p* < 0.05; *F* = 6.62, *d.f.* = 2,6, *p* < 0.05). In the waxy cultivar population, the egg-laying rate of the F3 generation on the original maize cultivar significantly differed from that of the F1 and F2 generations (*F* = 5.40, *d.f.* = 2,6, *p* < 0.05), whereas in the common cultivar population, the egg-laying rate of the F1 generation was significantly different from that of the F2 and F3 generations (*F* = 5.40, *d.f.* = 2,6, *p* < 0.05).

### 3.2. The Feeding Preferences of Spodoptera frugiperda Larvae on Four Maize Cultivars for Three Consecutive Generations of Feeding Experiences

**Figure 1:**
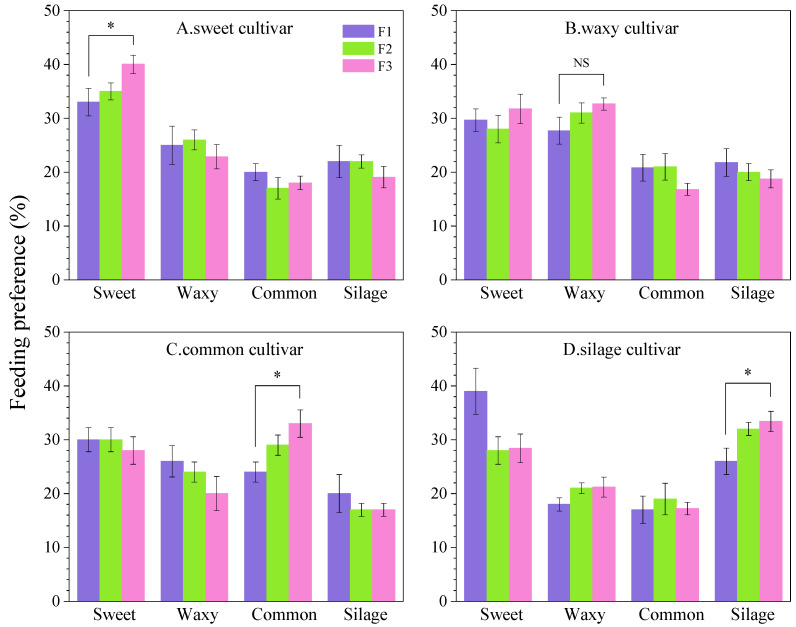
Feeding preferences of *Spodoptera frugiperda* larvae on 4 maize cultivars for three consecutive generations after 24 h. The data presented are mean ± SE. Statistical significance was assessed using the Duncan multiple comparison test. The error bar in the figure is SE. (* *p* < 0.05, NS *p* > 0.05).

The feeding preferences of FAW larvae populations continuously reared on the same maize cultivar for three generations exhibited different variations, either among different cultivars within the same generation or among different generations on the same cultivar (Figure 1). In all populations, the F1 generation larvae consistently demonstrated a feeding preference for the sweet maize cultivar. However, their preference for natal host plants gradually increases with successive generations. For example, populations reared on sweet, common, and silage cultivars showed significant differences in larval feeding preference for their natal maize cultivar in all generations (*p* < 0.05). Although no significant differences were observed among the three generations in the waxy cultivar population, the preference rate for natal hosts also exhibited an increasing trend with generational progression.

### 3.3. Effects of Different Feeding Experiences on the Developmental Duration of Spodoptera frugiperda

All FAWs were able to complete their life cycle when reared for multiple generations with the four maize cultivars (Table 2). In the common cultivar treatment, the larval stage (18.4 d), pupal stage (9.5 d), pre-adult stage (29.2 d), and total duration (37.4 d) were longer than those in the other three treatments. This indicates that FAWs feeding on the common cultivar developed relatively slowly.

### 3.4. Effects of Different Feeding Experiences on Pupal Weight of Spodoptera frugiperda

There were significant differences (*p* < 0.05) in the pupal weights of different populations when the FAW was reared with the four maize cultivars after three consecutive generations (Table 3). The pupal weight of both female and male insects was the highest on the sweet cultivar treatment (152.6 mg and 159.8 mg, respectively), and the lowest on the silage cultivar treatment (132.7 mg and 141.8 mg, respectively). The pupal weight of male moths in all treatments was higher than that of the females. With data pooled together, the pupal weight of the treatment on the sweet cultivar was still the heaviest at 155.9 mg, while the pupal weight of the treatment on the silage cultivar was the lightest at 137.3 mg.

**Table 3:** Pupal weight of *Spodoptera frugiperda* reared on the four maize cultivars.

Sex	Pupal Weight (mg)						
	Sweet Cultivar	Waxy Cultivar	Common Cultivar	Silage Cultivar	*F*	*d.f.*	*p*
Female	152.6 ± 2.2 a	143.1 ± 3.8 b	142.7 ± 3.8 b	132.7 ± 3.2 c	6.83	3,118	<0.05
Male	159.8 ± 3.8 a	152.6 ± 4.9 ab	155.8 ± 4.0 a	141.8 ± 3.9 b	3.50	3,109	<0.05
Total	155.9 ± 2.1 a	147.63 ± 3.1 b	149.2 ± 2.9 ab	137.3 ± 2.6 c	8.67	3,231	<0.05

The data in the table are presented as mean ± SE. (One-way ANOVA, Duncan test, *p* < 0.05.) Different letters indicate significant differences between the four cultivars of the same parameter.

### 3.5. Effects of Different Feeding Experiences on the Survival of Spodoptera frugiperda

The larval survival, pupation, and eclosion rates of the F3 generation of the FAW had different effects among the four treatments (Table 4). Compared with the other three treatments, the sweet cultivar had the highest cumulative survival, pupation, and emergence rates of larvae, which were 88.0%, 96.6%, and 85.7%, respectively. The silage cultivar treatment had the lowest cumulative survival and emergence rates of larvae, which were 83.0% and 82.1%, respectively. The waxy cultivar had the lowest pupation rate (90.5%).

**Table 4:** Survival rate of *Spodoptera frugiperda* reared on four maize cultivars.

Maize Cultivar	Survival Situation		
	Total Larval Survival Rate (%)	Pupation Rate (%)	Emergence Rate (%)
Sweet cultivar	88.0 ± 3.6 a	96.6 ± 2.0 a	85.7 ± 3.8 a
Waxy cultivar	84.0 ± 3.6 b	90.5 ± 3.2 d	85.5 ± 4.1 a
Common cultivar	84.0 ± 3.2 b	95.2 ± 2.3 b	85.0 ± 4.0 a
Silage cultivar	83.0 ± 3.5 c	94.0 ± 2.6 c	82.1 ± 4.4 b
*F*	41.97	98.37	22.06
*d.f.*	3,396	3,396	3,396
*p*	<0.05	<0.05	<0.05

The data in the table are presented as mean ± SE. (One-way ANOVA, paired bootstrap test, *p* < 0.05.) Different letters indicate significant differences between the four cultivars of the same parameter.

### 3.6. Effects of Different Feeding Experiences on the Reproduction of Adult Spodoptera frugiperda

The adult preoviposition period (APOP) of the FAW reared on the silage cultivar treatment was the longest at 4.4 d (Table 5). Compared with other treatments, the oviposition days (*O_d_*) of the sweet cultivar treatment was the longest (*O_d_* = 3.7), the mean fecundity of all female adults (*F*) and the mean fecundity of only reproductive female adults (*F_r_*) was the highest (*F* = 458 eggs/female, *F_r_* = 596 eggs/reproductive female), while the *O_d_* of the waxy cultivar treatment was the shortest (*O_d_* = 3.3 d), and the F of the silage cultivar treatment was the lowest (*F* = 371 eggs/female).

**Table 5:** Population parameters of *Spodoptera frugiperda* F1 and F3 generations reared on the four maize cultivars.

Parameters	F1 Generation				F3 Generation			
	Sweet Cultivar	Waxy Cultivar	Common Cultivar	Silage Cultivar	Sweet Cultivar	Waxy Cultivar	Common Cultivar	Silage Cultivar
Total larval duration (d)	17.4 ± 0.1 a	17.7 ± 0.2 a	18.5 ± 0.1 a	17.6 ± 0.2 a	16.8 ± 0.1 b	16.2 ± 0.1 b	16.8 ± 0.1 b	15.4 ± 0.1 b
Pupa (d)	10.5 ± 0.1 a	10.4 ± 0.1 a	10.3 ± 0.1 a	10.4 ± 0.1 a	8.7 ± 0.1 b	8.8 ± 0.2 b	9.5 ± 0.1 b	9.0 ± 0.2 b
Immature survival rate (*s_a_*) (%)	74 ± 4 a	70 ± 5 a	70 ± 5 a	67 ± 5 a	72 ± 4 a	65 ± 5 a	68 ± 5 a	64 ± 5 a
Mean longevity (d)	33.9 ± 1.0 a	32.8 ± 1.1 a	33.2 ± 1.2 a	32.0 ± 1.2 a	31.4 ± 1.0 a	29.8 ± 1.0 b	31.4 ± 1.0 a	28.6 ± 1.0 b
APOP (d)	4.3 ± 0.1 a	4.1 ± 0.2 a	4.2 ± 0.2 a	4.4 ± 0.2 a	3.5 ± 0.2 b	3.7 ± 0.2 a	3.4 ± 0.1 b	3.8 ± 0.1 b
TPOP (d)	34.3 ± 0.3 a	35.0 ± 0.4 a	35.4 ± 0.3 a	35.4 ± 0.4 a	31.5 ± 0.3 b	30.5 ± 0.3 b	31.9 ± 0.3 b	30.2 ± 0.3 b
Oviposition day (d)	3.7 ± 0.1 a	3.5 ± 0.2 a	3.0 ± 0.2 a	3.2 ± 0.2 a	3.7 ± 0.1 a	3.3 ± 0.2 a	3.3 ± 0.2 a	3.5 ± 0.1 a
F (all female) (eggs/female)	556 ± 28 a	531 ± 31 a	398 ± 31 a	399 ± 30 a	458 ± 46 a	382 ± 39 b	425 ± 44 a	371 ± 40 a
F_r_ (rep. female) (eggs/female)	603 ± 9 a	581 ± 16 a	458 ± 20 a	452 ± 19 b	596 ± 29 a	481 ± 26 b	520 ± 32 a	504 ± 17 a
*R*_0_ (offspring)	211.14 ± 29.01 a	185.78 ± 27.56 a	151.30 ± 22.53 a	135.53 ± 21.32 a	178.70 ± 28.66 a	129.98 ± 22.39 a	140.30 ± 24.56 a	125.97 ± 22.14 a
*r* (d^−1^)	0.15 ± 0.04 a	0.14 ± 0.04 a	0.14 ± 0.04 a	0.13 ± 0.05 b	0.15 ± 0.05 a	0.15 ± 0.06 a	0.15 ± 0.06 a	0.15 ± 0.06 a
*λ* (d^−1^)	1.16 ± 0.05 a	1.15 ± 0.05 a	1.15 ± 0.05 a	1.14 ± 0.05 b	1.17 ± 0.06 a	1.16 ± 0.07 a	1.16 ± 0.06 a	1.16 ± 0.07 a
*T* (d)	35.8 ± 0.3 a	36.5 ± 0.4 a	36.9 ± 0.3 a	36.8 ± 0.4 a	33.8 ± 0.2 b	32.4 ± 0.3 b	33.7 ± 0.3 b	32.1 ± 0.3 b

Different letters indicate significant differences between the F1 and F3 generations of the same host plant. The data in the table are presented as mean ± SE. (One-way ANOVA, paired bootstrap test, *p* < 0.05.) (APOP: the period between adult emergence and first oviposition. TPOP: the period between egg birth and adult first oviposition.)

### 3.7. Effects of Different Feeding Experiences on Life Table Population Parameters of Spodoptera frugiperda

The mean generation time (*T*) showed a significant difference (*p* < 0.05), whereas the net reproductive rate (*R*_0_), intrinsic rate of increase (*r*), and finite rate of increase (*λ*) were not significantly different (*p* > 0.05) among the FAW populations reared on the four maize cultivars (Table 5).

### 3.8. Parameters of the F1 and F3 Generations of Spodoptera frugiperda Feeding on Four Maize Cultivars

The larval stage, pupal stage, total preoviposition period (TPOP), and mean generation time (*T*) of the F3 generation of the FAW were significantly shorter than those of the F1 generation (*p* < 0.05, Table 5). The survival rate of the immature stage (*S_a_*) and the net reproductive rate (*R*_0_) of the F3 generation were similar to those of the F1 generation. The intrinsic rate of increase (*r*) and finite rate of increase (*λ*) in the sweet, waxy, common, and silage cultivars increased in the F3 generation, but only the increase in the silage cultivars was significant (*p* < 0.05). The fecundity per female (*F_r_*) and mean fecundity (*F*) of the ovipositing female insects on the waxy cultivar decreased as the number of generations increased, while those reared on the silage cultivar increased.

### 3.9. Age-Stage Survival Rate of Spodoptera frugiperda with Different Feeding Experiences

**Figure 2:**
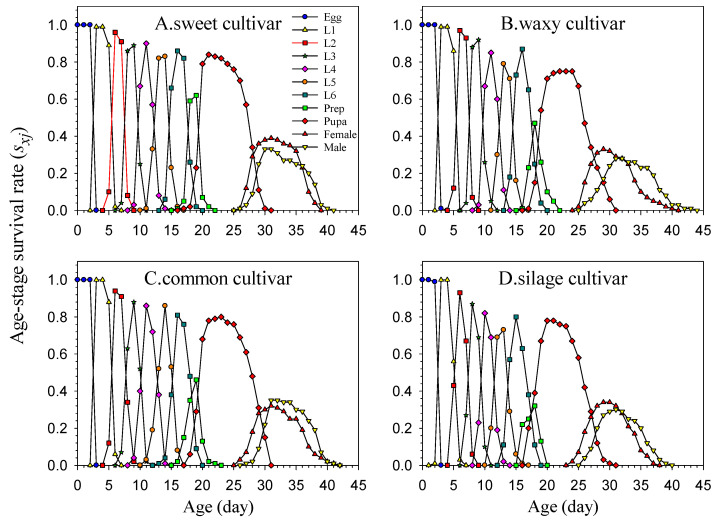
Age-stage survival rate (*s_xj_*) of *Spodoptera frugiperda* reared on four maize cultivars. Figure 2, Figure 3, Figure 4 and Figure 5 are the F3 data of FAW.

The survival rates (*s_xj_*) of FAW populations continuously reared on the four maize cultivars for three generations were relatively higher than those of the first generation (Figure 2). The survival rates of the pupal stage reared on the sweet, waxy, common, and silage cultivars were 85%, 78%, 80%, and 77%, respectively. At the initial adult stage, the survival rate of female moths was higher than that of males, whereas the results in the later stages were the opposite. Therefore, the total lifespan of the FAW on the waxy cultivar and common cultivar after three generations of rearing was longer than that on the sweet cultivar and silage cultivar populations.

### 3.10. Age-Specific Survival Rate and Population Fecundity of Spodoptera frugiperda with Different Feeding Experiences

**Figure 3:**
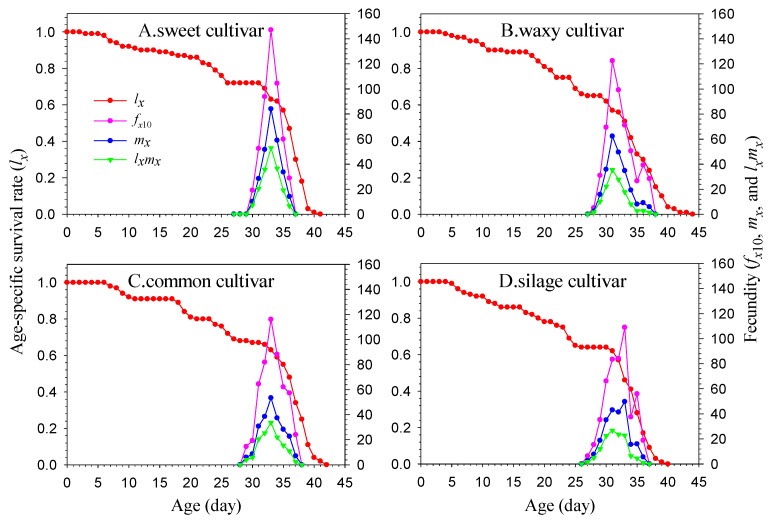
Age-specific survival rate (*l_x_*), female age-specific fecundity (*f_x_*_10_) (female adult is the 10th stage), age-specific fecundity (*m_x_*), and age-specific net maternity (*l_x_m_x_*) of *Spodoptera frugiperda* reared on the four maize cultivars.

Age-specific fecundity (*m_x_*) was the average number of eggs produced by the FAW population (Figure 3). During the entire development process, the female on the silage cultivar started to oviposit first, while on the common cultivar, it was the last to oviposit. The peak values of the mx curves are 85, 62, 57, and 52, respectively. The *m_x_* value for the sweet cultivar was the highest, whereas that of the silage cultivar was the lowest. The parameter *f_x_*_10_ is the average daily egg production at age *x* stage 10. Here, the sweet cultivar had the highest oviposition peak, whereas the silage cultivar had the lowest oviposition peak. Finally, *l_x_m_x_* is the total number of eggs laid per female at age x and can indicate the population fertility, considering the survival rate. The *l_x_m_x_* peak values for FAWs feeding on the sweet cultivar, waxy cultivar, common cultivar, and silage cultivar were 52.36, 37.16, 36.89, and 28.62, respectively, and the first time of reproduction on the sweet cultivar was the fastest among them.

### 3.11. The Expected Lifespan of the Population of Spodoptera frugiperda with Different Feeding Experiences

**Figure 4:**
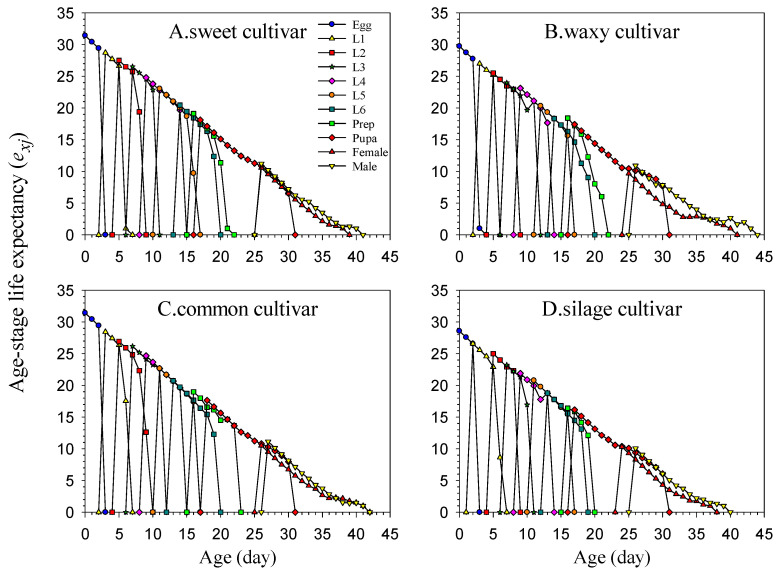
Population life expectancy (*e_xj_*) of *Spodoptera frugiperda* reared on four maize cultivars.

Age-stage-specific life expectancy (*e_xj_*) represents the expected lifespan of each individual (Figure 4). The expected value curve for the silage cultivar dropped the fastest, and the expected value of the starting lifespan at each stage was lower than that of other populations, indicating that the growth rate of the FAW on the silage cultivar was faster than that of other populations, which was the same as the result of the larval development duration on the silage cultivar. In addition, the expected lifespan values of male adults in the four FAW populations were higher than those of female adults.

### 3.12. The Population Reproductive Value of Spodoptera frugiperda with Different Feeding Experiences

**Figure 5:**
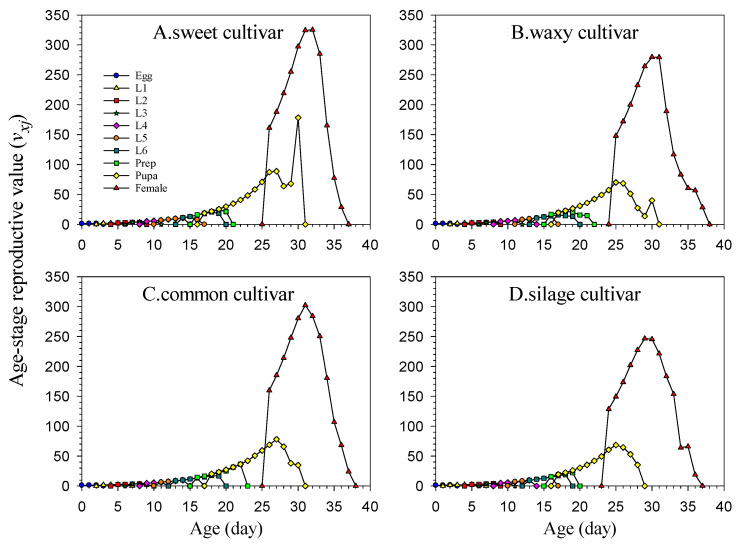
Population reproduction value (*v_xj_*) of *Spodoptera frugiperda* reared on four maize cultivars.

The age-stage-specific reproductive value (*v_xj_*) refers to the average contribution of an individual to future population growth (Figure 5). The reproductive value increased with the larval instar number. The initial egg-laying reproductive values of the FAW population reared on the sweet, waxy, common, and silage cultivars were 1.1656, 1.1623, 1.1578, and 1.1625, respectively, which were consistent with the finite rate of increase (*λ*) in the life table parameters. The reproductive peaks of the FAW population appeared at 32–34 days among the four treatments, which were (sweet cultivar, 32 days, 325 eggs/female), (waxy cultivar, 30 days, 280 eggs/female), (common cultivar, 31 days, 302 eggs/female), and (silage cultivar, 29 days, 246 eggs/female), respectively. The reproductive value peak of the sweet cultivar was the highest but appeared the latest, and the peak of the silage cultivar was the lowest but appeared the earliest. The egg-laying days of female adults on the sweet, waxy, common, and silage cultivars were 11, 13, 12, and 13 days, respectively. The peak reproductive value of female adults on the sweet cultivars was the highest, but the reproduction duration was the shortest.

## 4. Discussion

The results of this study revealed that F1 generation fall armyworm populations reared on the four maize cultivars exhibited larval and adult preferences for sweet maize. Relevant studies have shown that substrate characteristics, such as sweetness and hardness, influenced oviposition choices, with a positive correlation observed between oviposition preference and glucosinolate content in host plants, which may also explain the fall armyworm’s preference for sweet maize [29,30]. With successive generations, the preference hierarchy of the FAW for the four maize cultivars changed significantly and consistently as larvae and adults gradually increased their feeding and oviposition activities on their original host plants. This indicated that the FAW had learning behavior, and feeding experience could induce preference-driven host selection, aligning with Hopkins’ host selection principle (HHSP). Female onion flies (*Delia antiqua*) exhibit a strong preference and deposit more eggs on their original host plant [13]. Conversely, when female tobacco hornworms (*Manduca sexta* L.) completed a single oviposition event on one of two host plants (*Datura wrightii* or *Nicotiana attenuata*), they immediately selected the previously experienced plant foliage when re-exposed to both options, demonstrating that a single oviposition event can drive subsequent oviposition preference changes [31].

Host plants play an important role in the growth, development, and reproduction of insects, and suitable hosts can improve the growth rate, survival rate, and fecundity of insect offspring [32]. This experiment studied the effects of feeding on different maize cultivars on the growth, development, and reproduction of successive generations of FAW until the F3 generation. These results showed significant effects on the developmental duration of each insect stage, pupal weight, survival rate, and oviposition of the FAW population reared on the different maize cultivars. FAWs feeding on six rice cultivars resulted in significant differences in larval development duration, pupal duration, pupation rate, adult lifespan, and egg production [33]. These results showed that the larval stage and adult lifespan of FAWs reared on sweet maize were both longer than those reared on waxy maize [34]. Similarly, Zhang et al. indicated that special maize cultivars are more suitable for the growth and development of FAWs than ordinary maize cultivars [35]. Compared to common maize cultivars, these populations have stronger adaptability to waxy maize, which is consistent with the results of our study. However, the larval stage and adult lifespan of FAWs feeding on waxy maize are longer than those on common maize, which is contrary to the results of this study, and might be caused by differences in cultivars or rearing patterns [10,36]. Generally, when an insect on a certain host plant has higher fitness, it will have higher rates of development, survival, and reproduction. Although the growth and development speed are faster when the FAW feeds on wheat (*Triticum aestivum* L.) than on maize, its food utilization efficiency and population reproduction ability were all lower [37], which is similar to our results showing that the larval development duration of the silage maize population was the shortest, but the survival, pupation, and eclosion rates were the lowest.

The state of the pupa reflects the adaptability of the larva to a particular host or environment, and the weight of the pupae reflects the insect’s appetite for the host plant [38]. The pupal weight and fecundity of female lepidopteran adults are positively correlated with their adaptive potential [35]. The results of our study showed that the pupal weight of the FAW differed significantly among the four maize cultivars (sweet maize > common maize > waxy maize > silage maize). Meanwhile, the larval survival rate, pupal weight, and egg production of female adults of FAWs fed sweet maize were significantly higher than those fed waxy maize [34]. Additionally, the pupal weight and egg production of FAWs fed on common maize were both higher than those of waxy maize [10]. These results are consistent with those of our study. The female pupal weight on special maize cultivars was higher than that of common maize cultivars [35]; however, in our study, the pupal weight of waxy maize was lower than that of common maize, which may be related to the nutritional quality or resistance among different maize cultivars. Other relevant studies have shown that the change in pupal weight may be related to the amino acid content among different cultivars. Cultivars with strong resistance generally have higher glutamic acid content and lower tyrosine content, and the resulting pupal weight is lighter [39].

The biological parameters *R*_0_, *r*, *λ*, and *T* indicate the growth, development, reproduction, and survival changes in insects and the population growth ability in a specific environment [40]. There were significant differences in the population parameters of FAWs among different maize cultivars. Our results showed that the *R*_0_, *r*, and *λ* values were highest on sweet maize. Similarly, the intrinsic rate of increase and net reproductive rate of FAWs feeding on sweet maize were also higher than those on waxy maize [34]. In contrast, Zhang et al. reported that the net reproductive rate and intrinsic rate of growth were waxy maize > sweet maize > common maize, which was different from the results of our study [35]. This may be caused by different generations of FAW on the same host.

FAWs feeding on plants with secondary substances that are different from hosts for multiple generations will cause changes in the activities of some enzymes in the insects and have an impact on the growth and development of larvae [41]. When the FAW feeds on *Vicia villosa* Roth, as the number of generations increases, its adaptability gradually decreases until it cannot complete a subsequent generation or maintain its population at the fourth generation [22]. When returned to the original host maize, the offspring displayed reduced performance and loss of adaptation to their host. Although Bt cotton had a negative impact on *Spodoptera exigua* Hübner for three continuous generations, the survival rate and fecundity of adults increased significantly, and the lipase and trypsin activities of the third generation were significantly lower than those of the first generation; however, the activities of carboxylesterase and acetylcholinesterase were significantly higher than those of the first generation [42]. This indicates that the enhancement of its adaptability may be closely related to the enhancement of detoxifying enzyme activities rather than digestive enzymes. Therefore, further verification is needed in the next research. Similarly, the pupal weight, survival rate, fecundity, relative growth rate, and relative digestion rate of the third generation were all significantly higher than those of the first generation when the larvae of *Helicoverpa armigera* Hübner fed on high-gossypol cultivars [21]. The developmental duration was prolonged with an increase in successive rearing generations when the guava fruit fly (*Bactrocera correcta* Bezzi) was continuously reared indoors on an artificial diet [43]. After being fed rape pollen, the F1 and F2 generations of *Micraspis discolor* showed a lower survival rate and female ratio, but the F3 and F4 generations had higher survival rates, female ratios, and weights, suggesting that *M. discolor* gradually adapted to the pollen [44].

Developmental duration is critically important for insect survival, as prolonged exposure to natural environments elevates the risk of biotic (e.g., pathogens and natural enemies) and abiotic (e.g., natural disasters and adverse environmental conditions) stressors, which may pose critical threats to their viability. Because the life table of the F1 generation of FAWs has already been studied in our previous experiments, we measured the parameters of the F3 generation in our study. By comparing our results with those of the F1 generation, it can be seen that the developmental duration of the F3 generation of FAWs was shortened significantly [45]. This indicates that the FAW can adapt to diverse plant species through evolution, shortened developmental duration, and mitigated adverse factors affecting its development, thereby enhancing its adaptability to different ecological regions.

In conclusion, since FAWs are a type of polyphagous pest that can damage different crops and cultivars within a certain area, at the same time, long-term cultivation of a single plant or cultivar may also aggravate the damage situation of FAWs. Therefore, it is necessary to implement habitat regulation technology such as intercropping instead of a single crop or variety in the field, or plant protective row plants at the edge of the field, to reduce the possibility of multiple generations of FAWs completing their development on the same plant. These strategies will reduce the loss of crop production due to the presence of FAWs.

## Figures and Tables

**Table 1:** Oviposition preference (mean ± SE) of *Spodoptera frugiperda* adults on four maize cultivars for three consecutive generations.

Population	Generation	Maize Variety			
		Sweet Cultivar	Waxy Cultivar	Common Cultivar	Silage Cultivar
	F1	26.5 ± 0.4 b	24.9 ± 1.2 a	23.6 ± 1.0 a	25.0 ± 0.3 a
Sweet cultivar	F2	28.7 ± 0.9 ab	24.3 ± 0.4 a	21.5 ± 0.3 a	25.5 ± 0.5 a
	F3	31.3 ± 1.4 a	22.9 ± 1.2 a	21.5 ± 0.7 a	24.3 ± 0.8 a
	F1	28.0 ± 1.1 a	23.2 ± 0.1 b	24.0 ± 0.9 a	24.8 ± 0.6 a
Waxy cultivar	F2	27.0 ± 1.0 a	23.8 ± 0.6 b	24.0 ± 0.8 a	25.2 ± 0.5 a
	F3	26.8 ± 0.8 a	26.2 ± 1.0 a	23.9 ± 1.8 a	23.2 ± 1.0 a
	F1	27.8 ± 1.4 a	22.8 ± 0.7 a	25.2 ± 0.8 b	24.3 ± 0.3 a
Common cultivar	F2	26.3 ± 0.9 a	22.8 ± 0.9 a	28.1 ± 0.8 a	22.8 ± 1.2 a
	F3	27.0 ± 1.7 a	21.4 ± 1.1 a	28.2 ± 0.6 a	23.5 ± 1.9 a
	F1	28.1 ± 2.0 a	22.7 ± 0.6 a	23.7 ± 1.0 a	25.5 ± 1.0 b
Silage cultivar	F2	25.7 ± 0.3 a	22.2 ± 0.1 a	24.6 ± 0.7 a	27.5 ± 0.8 ab
	F3	24.1 ± 0.5 a	21.7 ± 1.4 a	23.7 ± 0.7 a	30.5 ± 1.1 a

Different letters indicate significant differences in egg attachment rates among the F1, F2, and F3 generations of the same *Spodoptera frugiperda* population on the same maize cultivar (one-way ANOVA, Duncan test, *p* < 0.05).

**Table 2:** Development time, adult longevity, and total longevity of *Spodoptera frugiperda* reared on four maize cultivars.

Developmental Stage	Maize Cultivar							
	*n*	Sweet Cultivar	n	Waxy Cultivar	*n*	Common Cultivar	*n*	Silage Cultivar
Egg (d)	99	3.0 ± 0.0 a	99	3.0 ± 0.0 a	100	3.0 ± 0.0 a	100	3.0 ± 0.0a
1st instar (d)	96	2.9 ± 0.1 a	97	2.8 ± 0.0 a	98	2.9 ± 0.1 a	94	2.6 ± 0.1 b
2nd instar (d)	92	2.1 ± 0.0 b	95	2.0 ± 0.0 b	93	2.3 ± 0.0 a	93	2.2 ± 0.1 a
3rd instar (d)	91	2.2 ± 0.9 a	90	2.2 ± 0.1 a	91	2.3 ± 0.1 a	90	2.0 ± 0.1 b
4th instar (d)	90	2.5 ± 0.1 b	89	2.5 ± 0.0 ab	91	2.7 ± 0.1 a	86	2.1 ± 0.1 c
5th instar (d)	88	2.5 ± 0.0 a	89	2.2 ± 0.0 c	91	2.4 ± 0.1 ab	86	2.3 ± 0.0 bc
6th instar (d)	87	3.1 ± 0.1 a	84	3.0 ± 0.1 a	84	2.8 ± 0.1 b	83	3.0 ± 0.1 a
Total larval duration (d)	87	15.2 ± 0.1 a	84	15.0 ± 0.1 b	84	15.4 ± 0.1 a	83	14.3 ± 0.1 c
Prepupa (d)	84	1.2 ± 0.1 c	76	1.5 ± 0.1 a	80	1.4 ± 0.1 b	78	1.4 ± 0.1 b
Pupa (d)	72	8.7 ± 0.2 b	65	8.8 ± 0.1 b	68	9.5 ± 0.1 a	64	9.0 ± 0.1 a
Total immature duration (d)	72	28.1 ± 0.2 b	65	28.3 ± 0.2 b	68	29.2 ± 0.2 a	64	27.7 ± 0.2 c
Immature survival rate (*s_a_*) (%)	100	72 ± 4 a	100	65 ± 5 a	100	68 ± 5 a	100	64 ± 5 a
Female adult longevity (d)	39	8.5 ± 0.3 a	34	7.8 ± 0.4 a	33	8.2 ± 0.4 a	34	7.8 ± 0.3 a
Male adult longevity (d)	33	8.4 ± 0.4 a	31	7.6 ± 0.4 a	35	8.1 ± 0.3 a	30	7.2 ± 0.3 a
Female total longevity (d)	39	36.6 ± 0.3 a	34	34.7 ± 0.5 b	33	36.6 ± 0.5 a	34	34.3 ± 0.3 b
Male total longevity (d)	33	37.2 ± 0.4 a	31	37.4 ± 0.6 ab	35	38.1 ± 0.3 a	30	36.1 ± 0.4 b
Mean longevity (all individuals) (d)	100	31.4 ± 1.0 a	100	29.8 ± 1.0 ab	100	31.4 ± 1.0 a	100	28.6 ± 1.0 b

The data in the table are presented as mean ± SE. Different letters in the same row indicate significant differences among different host plants (one-way ANOVA, paired bootstrap test, *p* < 0.05). Table 2, Table 3 and Table 4 are the F3 data of FA.

## Data Availability

The original contributions presented in this study are included in the article. Further inquiries can be directed to the corresponding author.

## Appendix A

**Table A1:** Artificial diet formulation.

Component	Quantity
Wheat germ powder	280 g
Soy protein powder	90 g
Yeast powder	35 g
Agar	25 g
Vitamin B complex	0.2 g
Cholesterol	12 g
Sorbic acid	2 g
Ascorbic acid	12 g
Methyl p-hydroxybenzoate	5 g
Formaldehyde	4 mL
Penicillin	0.2 g
Distilled water	1500 mL

**Table A2:** The definition, equation, and reference of population parameters used in this study.

Parameter	Equation	Definition and Reference
Age-specific survival rate (*l_x_*)	$l_{x}=\sum_{j=1}^{m}s_{xj}\;$	The probability that a newborn offspring survives to age *x.* It includes female, male, and those that died in the pre-adult stages; *m* is the number of stages [40].
Age-specific fecundity (*m_x_*)	$m_{x}=\sum_{j=1}^{m}s_{xj}f_{xj}/\sum_{j=1}^{m}s_{xj}$	The mean fecundity of individuals at age *x* [40].
Net reproductive rate (*R*_0_) and cumulative net reproductive rate (*R_x_*)	$R_{0}=\sum_{x=0}^{\infty }l_{x}m_{x},R_{x}=\sum_{i=0}^{x}l_{i}m_{i}$	*R*_0_ is the total offspring that an average individual (including females, males, and those that died in the pre-adult stage) can produce during its lifetime [40]. *R_x_* is the total offspring that an average individual can produce from age 0 to age *x*.
Intrinsic rate of increase (*r*)	$\sum_{x=0}^{\infty }e^{-r\left(x+1\right)}l_{x}m_{x}=1$	The population growth rate as time approaches infinity and the population reaches the stable age-stage distribution (SASD). It is calculated by using the Euler–Lotka equation with age indexed from 0 [40].
The finite rate of increase (*λ*)	$\lambda =e^{r}$	The population growth rate as time approaches infinity and the population reaches a stable age-stage distribution. The population size will increase at the rate of *λ* per unit time [40].
Mean generation time (*T*)	$T=\ln R_{0}/r$	The time length that a population requires to increase to *R*_0_-fold of its size as time approaches infinity and the population settles down to a stable age-stage distribution [40].
Age-stage life expectancy (*e_xj_*)	$e_{xj}=\sum_{i=x}^{\infty }\sum_{y=j}^{m}{s^{\prime }}_{iy}\;$	The time length that an individual of age *x* and stage *j* is expected to live. The notation ${s^{\prime }}_{iy}$ is the probability that an individual of age *x* and stage *j* will survive to age *i* and stage *y*. It is calculated according to Chi and Liu (1985) by assuming *s_xj_* = 1 [26,40].
Age-stage reproductive value (*v_xj_*)	$v_{xj}=\frac{e^{r(x+1)}}{s_{xj}}\sum_{i=x}^{\infty }e^{-r\left(x+1\right)}\sum_{y=j}^{m}{s^{\prime }}_{iy}f_{iy}$	The contribution of an individual of age *x* and stage *j* to the future population [27].
